# Endovascular Treatment for Acute Basilar Artery Occlusion: A Fragility Index Meta-Analysis

**DOI:** 10.3390/jcm12072617

**Published:** 2023-03-30

**Authors:** Lina Palaiodimou, Andreas Eleftheriou, Aristeidis H. Katsanos, Apostolos Safouris, Georgios Magoufis, Stavros Spiliopoulos, Georgios Velonakis, Sofia Vassilopoulou, Diana Aguiar de Sousa, Guillaume Turc, Daniel Strbian, Georgios Tsivgoulis

**Affiliations:** 1 Second Department of Neurology, “Attikon” University Hospital, School of Medicine, National and Kapodistrian University of Athens, 15784 Athens, Greece; 2 Division of Neurology, McMaster University and Population Health Research Institute, Hamilton, ON L8L2X2, Canada; 3 Stroke Unit, Metropolitan Hospital, 18547 Piraeus, Greece; 4 Aktios Rehabilitation Center, 15341 Athens, Greece; 5 Department of Neurointerventions, National Institute of Mental Health, Neurology and Neurosurgery, Section of Neurointervention, Department of Neurosurgery, Semmelweis University, 1085 Budapest, Hungary; 6 Interventional Radiology Department, “Attikon” University Hospital, 12462 Athens, Greece; 7 Research Unit of Radiology, 2nd Department of Radiology, Eginition Hospital, National and Kapodistrian University of Athens, 15784 Athens, Greece; 8 First Department of Neurology, Eginition Hospital, School of Medicine, National and Kapodistrian University of Athens, 15784 Athens, Greece; 9 Stroke Center, Lisbon Central University Hospital, 1649-024 Lisbon, Portugal; 10 Institute of Anatomy and CEEM, Faculdade de Medicina, Universidade de Lisboa, 1649-004 Lisbon, Portugal; 11 Department of Neurology, GHU Paris Psychiatrie et Neurosciences, 75014 Paris, France; 12 Department of Neurology, Faculty of Medicine, Université Paris Cité, 75006 Paris, France; 13 INSERM U1266, 75014 Paris, France; 14 FHU NeuroVasc, 75010 Paris, France; 15 Department of Neurology, Helsinki University Hospital and University of Helsinki, 00029 Helsinki, Finland; 16 Department of Neurology, University of Tennessee Health Science Center, Memphis, TN 38163, USA

**Keywords:** acute ischemic stroke, acute basilar artery occlusion, endovascular treatment, mechanical thrombectomy, basilar stroke, posterior circulation, meta-analysis, randomized controlled clinical trials

## Abstract

Introduction: High-quality evidence regarding the use of endovascular treatment (EVT) in patients with acute basilar artery occlusion (BAO) has been provided by recently completed randomized controlled clinical trials (RCTs). Methods: We conducted a systematic review and meta-analysis including all available RCTs that investigated efficacy and safety of EVT in addition to best medical treatment (BMT) versus BMT alone for BAO. The random-effects model was used, while the fragility index (FI) was calculated for dichotomous outcomes of interest. Results: Four RCTs were included comprising a total of 988 patients with acute BAO (mean age: 65.6 years, 70% men, median NIHSS: 24, 39% pretreatment with intravenous thrombolysis). EVT was related to higher likelihood of good functional outcome (RR: 1.54; 95% CI: 1.16–2.05; I^2^ = 60%), functional independence (RR: 1.83; 95% CI: 1.08–3.08; I^2^ = 79%) and reduced disability at 3 months (adjusted common OR: 1.96; 95% CI: 1.26–3.05; I^2^ = 59%) compared to BMT alone. Despite that EVT was associated with a higher risk for symptomatic intracranial hemorrhage (RR: 7.78; 95% CI: 2.36–25.61; I^2^ = 0%) and any intracranial hemorrhage (RR: 2.85; 95% CI: 1.50–5.44; I^2^ = 16%), mortality at 3 months was lower among patients that received EVT plus BMT versus BMT alone (RR: 0.76; 95% CI: 0.65–0.89; I^2^ = 0%). However, sufficient robustness was not evident in any of the reported associations (FI < 10) including the overall effect regarding the primary outcome. The former associations were predominantly driven by RCTs with recruitment limited in China. Conclusions: EVT combined with BMT is associated with a higher likelihood of achieving good functional outcomes and a lower risk of death at 3 months compared to BMT alone, despite the higher risk of sICH. An individual-patient data meta-analysis is warranted to uncover and adjust for potential sources of heterogeneity and to provide further insight.

## 1. Background

Endovascular treatment (EVT) for acute ischemic stroke (AIS) attributed to large vessel occlusion (LVO) of the anterior circulation has been established as a highly efficient and safe treatment option [1]. It is considered as the standard of care for those patients, presenting a number needed to treat that ranges from 3 to 10 for achieving a good or excellent functional outcome at 3 months [2]. Current guidelines, both by the European Stroke Organisation (ESO) and the American Heart Association/American Stroke Association (AHA/ASA), recommend offering EVT to AIS patients with LVO in the anterior circulation, by using either mechanical thrombectomy or thrombus aspiration or a combination of those [3,4]. EVT may also be considered in anterior circulation LVO patients presenting in the prolonged time window (beyond 6 h from symptom onset), if they fulfill certain neuroimaging criteria in the form of either perfusion mismatch or clinical/imaging mismatch based on two landmark trials [5,6]. However, specific guidance regarding EVT in AIS patients with LVO of the posterior circulation and, more specifically, those with acute basilar artery occlusion (BAO), is limited to expert opinions [3]. Yet, acute BAO has been associated with a higher likelihood of dependency and mortality at 3 months compared to AIS patients with LVO in the anterior circulation [7,8].

Observational data suggest that EVT for acute BAO may be efficient and safe [9]. However, the randomized controlled clinical trials (RCTs) until 2022 have been inconclusive and do not support these observational findings [9]. Both “Basilar Artery International Cooperation Study” (BASICS) [10] and “Acute basilar artery occlusion: Endovascular Interventions versus Standard Medical Treatment” (BEST) [11] trials showed that EVT was not associated with better clinical outcomes, while there was a higher risk of symptomatic intracranial hemorrhage (sICH) compared to patients that received best medical treatment (BMT) alone. During 2022, the results of two other RCTs were made available: the “Endovascular Treatment for Acute Basilar Artery Occlusion” (ATTENTION) [12] and the “Basilar Artery Occlusion Chinese Endovascular Trial” (BAOCHE) [13] trials were able to detect a significantly higher rate of functional outcome at 3 months among patients that received EVT plus BMT versus those who were treated by BMT alone.

Given those inconsistent results of the RCTs published thus far, we performed a systematic review and study-level fragility index meta-analysis, with the aim to evaluate the efficacy and safety of EVT in patients with acute BAO.

## 2. Methods

### 2.1. Standard Protocol Approvals, Registrations, and Patient Consents

The pre-specified protocol of the present systematic review and meta-analysis has been registered in the International Prospective Register of Ongoing Systematic Reviews PROSPERO (registration ID: CRD42022373387) and is reported according to the updated Preferred Reporting Items for Systematic Reviews and Meta-Analyses (PRISMA) guidelines [14]. No ethical board approval or written informed consent by the patients were required due to the study design (systematic review and meta-analysis).

### 2.2. Data Sources, Searches and Study Selection

Following the PICO criteria, a systematic literature search was conducted to identify available RCTs evaluating adult patients with acute BAO within the last 24 h (P: population) treated with EVT together with BMT (I: intervention) versus BMT alone (C: comparator). Functional status at 3 months (O: outcome) should have been reported by the studies in order to be considered amenable for inclusion. EVT was defined as mechanical thrombectomy or endovascular aspiration or intra-arterial thrombolysis or a combination of those. BMT could also include intravenous thrombolysis (IVT) when administered to eligible patients. Two reviewers (LP and AE) independently searched MEDLINE and Scopus using the following terms: “acute basilar occlusion” and “endovascular treatment”; the complete search algorithms can be found in Methods S1 (Supplementary Materials). No language restrictions were applied. Final search was conducted on 29 November 2022. Reference lists of published articles were additionally searched manually to ensure the comprehensiveness of the literature search.

Non-controlled studies and any kind of observational study (i.e., cohort studies, case series or case reports), as well as editorials, commentaries and narrative reviews were discarded. All eligible studies were assessed by the two reviewers (L.P. and A.E.), and any disagreements were discussed and resolved by the corresponding author (G.T.).

### 2.3. Quality Control, Bias Assessment and Data Extraction

Quality control and bias assessment of eligible studies were performed independently by the two reviewers (L.P. and A.E.), employing the Cochrane Collaboration tool (RoB 2) for RCTs [15]. In case of conflicting opinions, these were settled by consensus after consulting with the corresponding author (G.T.).

Data were predominantly extracted by scrutinizing the peer-reviewed publications of the included RCTs along with their supplementary materials. In case of missing information, data were also sought in the presentations of the respective RCTs during international conferences. Data of interest (i.e., study name, period of enrolment, country, inclusion criteria, sample size and characteristics of included patients, outcomes) were extracted in structured reports.

### 2.4. Outcomes

The primary outcome of interest was the likelihood of achieving a good functional outcome at 3 months, as defined by a modified Rankin scale (mRS) score of 0 to 3, among patients treated with EVT and BMT versus BMT alone.

Secondary outcomes of interest comprised the following: (i) functional independence at 3 months as defined by a mRS of 0 to 2; (ii) reduced disability as assessed by ≥1-point reduction across all mRS grades at 3 months (shift analysis); (iii) sICH; (iv) any ICH; and (v) all-cause mortality at 3 months.

Both primary and secondary outcomes of interest were assessed in subgroup analyses stratified by recruiting centers (centers limited to China versus international participation). Furthermore, a second prespecified subgroup analysis stratifying for IVT pretreatment as part of BMT was also conducted with regard to the primary outcome.

### 2.5. Statistical Analysis

For each dichotomous outcome of interest, the corresponding risk ratio (RR) with 95% confidence interval (95% CI) was calculated using the random-effects model (DerSimonian and Laird) [16], comparing outcome events among patients treated with EVT versus controls. The subgroup differences between different distribution of recruiting centers were assessed by the Q test for subgroups [17]. For the shift mRS analysis, the adjusted common odds ratio (OR) was calculated using generic inverse variance meta-analysis. Pooled proportion of sICH was also calculated, after the implementation of the variance-stabilizing double arcsine transformation. The I^2^ and Cochran Q statistics were used for the assessment of heterogeneity; I^2^ values > 50% and values > 75% were considered to indicate substantial and considerable heterogeneity, respectively, while the significance level for the Q statistic was set at 0.1. Funnel plot inspection and the Egger’s linear regression test [18] were employed for the evaluation of publication bias, when more than four studies were included in the analysis of each outcome. Furthermore, the fragility index (FI) was calculated for the dichotomous outcomes of interest [19], with a FI ≤ 4 indicating a “highly fragile/non-robust” result; a 4 < FI ≤ 12 pointing to a somewhat “fragile/robust” result; a 12 < FI ≤ 34 corresponding to a “robust” result; and, finally, a FI > 34 suggesting a “highly robust” result [20]. All statistical analyses were performed using the Cochrane Collaboration’s Review Manager (RevMan 5.3) Software Package (Copenhagen: The Nordic Cochrane Centre, The Cochrane Collaboration, 2014) [21], and the OpenMetaAnalyst [22].

## 3. Results

### 3.1. Literature Search and Included Studies

**Figure 1:**
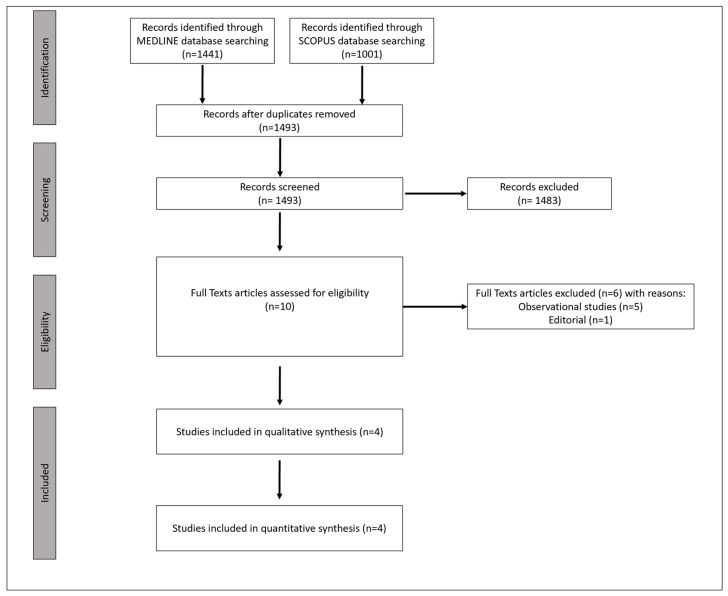
Flow chart of the systematic review.

Through a systematic database search (Figure 1, Table S1), four studies [10,11,12,13] were identified as eligible for inclusion in the systematic review and meta-analysis, with a total of 988 patients with acute BAO (mean age: 65.6 years; 70% men; median National Institutes Health Stroke Scale (NIHSS): 24; 39% IVT pretreatment; Figures S1–S4).

The characteristics of the included studies are presented in Table 1. Patient recruitment was limited to centers located in China in three of the included RCTs [11,12,13], while only one RCT had international recruitment [10]. Furthermore, two RCTs included patients that presented in the prolonged time window post symptom onset [12,13]. However, those two studies had more restrictive inclusion criteria, since patients were selected for participation according to a prespecified combination of age, pre-stroke mRS, baseline NIHSS, and Posterior Circulation Acute Stroke Prognosis Early CT Score (PC-ASPECTS) score [12,13]. This fact explains the differences noted in the baseline characteristics among patients of the different studies. Finally, patients and investigators were not blinded in any of the included studies. Yet, the evaluation of outcomes was performed by blinded assessors.

### 3.2. Quality Control of Included Studies

The quality assessment of the included studies is presented in Figures S5 and S6. All studies presented major concerns due to the fact that randomized participants and treating physicians were aware of the intervention and several deviations from intended interventions were noted. Moreover, one study [12] suffered from minor randomization bias since the use of a minimization process to balance the two treatment groups with appropriate stratification was not clearly reported, and another study [13] presented minor concerns due to missing outcome data. Overall, the included RCTs were considered of medium quality, mostly driven by the existence of performance bias.

### 3.3. Quantitative Analyses

An overview of analyses for all primary and secondary outcomes is summarized in Table 2.

### 3.4. Primary Outcome

**Figure 2:**
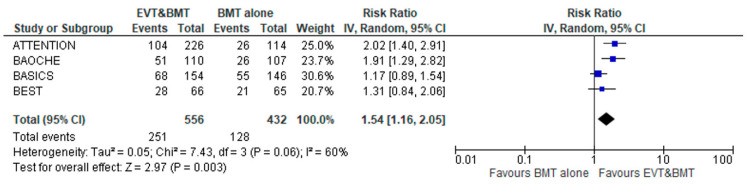
Forest plot presenting the association of endovascular treatment (EVT) combined with best medical treatment (BMT) compared to BMT alone with good functional outcomes (mRS 0–3) at 3 months among acute basilar artery occlusion patients.

Patients receiving EVT plus BMT had an increased likelihood of achieving good functional outcomes at 3 months compared to BMT alone (RR: 1.54; 95% CI: 1.16–2.05; 4 studies; I^2^ = 60%; *p* for Cochran Q = 0.06; Figure 2). However, FI was calculated at 9, indicating that the result was “fragile/somewhat robust”. In order to assess for potential reasons of the heterogeneity noted in this analysis, a subgroup analysis was conducted after stratification for the contributing centers (limited in China versus international recruitment). In this analysis, significant subgroup differences emerged (*p* for subgroup differences = 0.03), while the heterogeneity was also mitigated within the subgroups (Figure S7). When subgroup analysis was performed after stratification for IVT administration, no significant subgroup differences were noted either by RR (Figure S8) or by OR generic calculation (Figure S9), according to the effect size that was reported by the included studies. Notably, in the subgroup of patients pretreated with IVT, the effect size of EVT compared to BMT with regard to the primary outcome was attenuated (RR: 1.36; 95% CI: 1.05–1.77; I^2^ = 0%; *p* for Cochran Q = 0.50; data available from two RCTs).

### 3.5. Secondary Outcomes

EVT was also associated with higher likelihood of achieving functional independence at 3 months compared to BMT alone (RR: 1.83; 95% CI: 1.08–3.08; four studies; I^2^ = 79%; *p* for Cochran Q = 0.002; Figure S10). The FI was calculated at 4, which was indicative of a “highly fragile/non-robust” result. Furthermore, subgroup differences after stratification for recruiting centers were also evaluated and found to be marginally non-significant (*p* for subgroup differences = 0.07; Figure S11).

The adjusted common OR for reduced disability with EVT was 1.96 (95% CI: 1.26–3.05; four studies; I^2^ = 59%; *p* for Cochran Q = 0.06; Figure S12). No subgroup differences emerged among studies with recruiting centers limited to China or with international participation (*p* for subgroup differences = 0.20; Figure S13).

**Figure 3:**
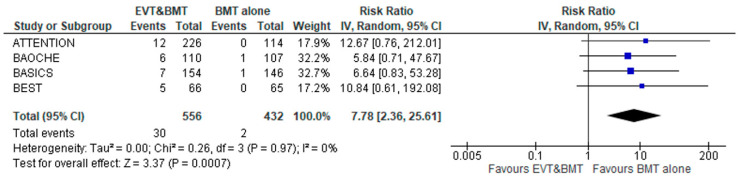
Forest plot presenting the association of endovascular treatment (EVT) combined with best medical treatment (BMT) compared to BMT alone with symptomatic intracranial hemorrhage (sICH) among acute basilar artery occlusion patients.

sICH was more common in the patients receiving EVT plus BMT versus BMT alone (RR: 7.78; 95% CI: 2.36–25.61; four studies; I^2^ = 0%; *p* for Cochran Q = 0.97; Figure 3). However, the result was considered “fragile/somewhat robust”, presenting a FI of 5. This may be attributed to the low frequency of sICH events in both arms. Notably, the pooled proportion of sICH in the interventional arm was calculated at 5.4% (95% CI: 3.6–7.4%; four studies; I^2^ = 0%; *p* for Cochran Q = 0.859; Figure S14). Stratified by recruiting centers, there was no difference between studies limited to China versus those with international recruitment with regard to sICH (*p* for subgroup differences = 0.86; Figure S15).

Any ICH was recorded more frequently in the interventional versus the control arm (RR: 2.85; 95% CI: 1.50–5.44; four studies; I^2^ = 16%; *p* for Cochran Q = 0.31; Figure S16). Yet, this was another “fragile/somewhat” robust result, with a FI of 5. No subgroup differences were noted among studies with limited versus international recruitment (*p* for subgroup differences = 0.13; Figure S17).

**Figure 4:**
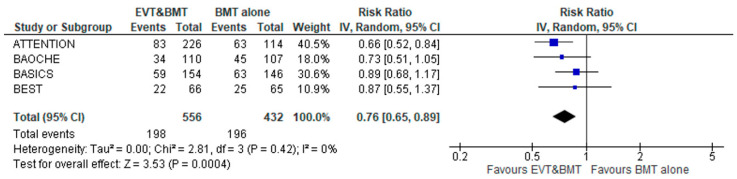
Forest plot presenting the association of endovascular treatment (EVT) combined with best medical treatment (BMT) compared to BMT alone with mortality at 3 months among acute basilar artery occlusion patients.

Finally, all-cause mortality at 3 months was significantly lower among the patients that were treated with EVT plus BMT versus BMT alone (RR: 0.76; 95% CI: 0.65–0.89; four studies; I^2^ = 0%; *p* for Cochran Q = 0.42; Figure 4). FI was calculated at 9, indicating a “fragile/somewhat robust” result. There were no subgroup differences among studies conducted in China versus those with international participation (*p* for subgroup differences = 0.19; Figure S18).

Evaluation for publication bias was not performed, since only four studies were included in the analysis.

## 4. Discussion

The present meta-analysis has shown that EVT plus BMT in the treatment of patients with acute BAO is associated with a higher likelihood of achieving a good functional outcome at 3 months compared to BMT alone, which rarely included IVT. Likewise, functional independence and any functional improvement at 3 months were also more common in the EVT plus BMT group versus the BMT alone group. Even though sICH and any ICH were observed more frequently in the interventional arm, the all-cause mortality at 3 months was significantly lower in the patients receiving EVT on top of BMT rather than BMT alone.

Despite the fact that statistical significance was achieved in the analysis for every outcome, the reported associations were not robust. This was evident by the relevantly low FI as assessed for every outcome and the heterogeneity noted, especially for the primary outcome of interest. To address the heterogeneity, a subgroup analysis stratified for the distribution of recruiting centers was conducted. There were statistically significant subgroup differences between studies that were conducted in China compared to international recruitment with regard to the primary outcome of good functional outcome at 3 months. It appears that the overall result of the meta-analysis is driven by the RCTs recruiting patients from China, and this fact may raise some concerns regarding the generalizability of the findings to international cohorts.

Given the important limitation that IVT is not directly reimbursed in China and the possibility that a subset of patients could not receive IVT despite indications [23], we hypothesized that the association between EVT and good functional outcome at 3 months would be amplified among patients that did not receive IVT as part of BMT. Characteristically, when stratification for contributing sites was performed, significant differences were disclosed with regard to IVT pretreatment: among the RCTs conducted in China, the proportion of IVT pretreatment was lower (26%; 95% CI: 17–36%) compared to the RCT with international participation (79%; 95% CI: 74–83%; *p* for subgroup differences < 0.001; Figure S4). Therefore, a second subgroup analysis was conducted specifically for the primary outcome of interest to investigate for potential differences among patients that received IVT as part of the BMT or not. After analysis of the available extracted data, IVT did not appear to significantly moderate the association of EVT with good functional outcome at 3 months. However, EVT compared to BMT did not appear to increase the rates of good functional outcome when the available ORs were pooled from three out of 4fourincluded RCTs (Figure S9). A recently published meta-analysis of observational data comparing EVT plus IVT versus EVT alone in acute BAO patients showed better functional outcomes in the combined treatment group without a significant increase in sICH [24]. Furthermore, observational data from a high-volume center showed that BAO patients achieved a 3-month mRS of 0–3 in about 45% when treated by IVT only [25], which is identical to the proportions presented in the EVT groups among included RCTs. Regarding further research, direct EVT versus bridging therapy in the treatment of BAO is the matter of investigation in the BEST-BAO trial, which will soon start recruitment in China (Direct Endovascular Treatment Versus Bridging Treatment In Basilar Artery Occlusive Stroke; https://www.clinicaltrials.gov; accessed on 29 November 2022; Unique identifier: NCT05631847).

Intracranial atherosclerosis is over-represented as a cause of AIS among RCTs conducted in the Chinese population, and this may act as another limitation to the generalizability of the results. Large artery atherosclerosis as the cause of AIS was 44% in the ATTENTION trial, 66% in the BAOCHE trial and 53% in the BEST trial [11,12,13]. By comparison, in the BASICS international trial, 34% of patients had AIS due to large artery atherosclerosis [10]. This discrepancy may have multifaceted treatment implications. First, atherosclerotic BAO is associated with worse prognosis [26], and atherosclerotic LVO is a therapeutic challenge that frequently necessitates angioplasty, stenting and intravenous antithrombotic medications in the acute phase that may result in hemorrhagic complications [27]. The site of occlusion is also linked to the underlying etiology, since embolic stroke will more frequently involve the distal basilar artery, whereas proximal BAO is usually linked to atherosclerotic lesions of the vertebrobasilar junction. These differences also have treatment implications, since it has been shown that recanalization rates are higher in patients with distal BAO occlusions [28]. Furthermore, among all stroke subtypes, adjunctive pharmacotherapy, such as antiplatelet agents and anticoagulants, either in the acute–subacute phase or as secondary stroke prevention measures, may inherently influence the efficacy and safety following acute reperfusion therapies [29,30].

Regarding safety outcomes, sICH was more common in the interventional arm compared to controls. The pooled proportion of sICH among patients that received EVT (5.4%) may have been higher than the one reported by the HERMES collaboration (3.8%) [31], but considering that a significant proportion of included patients were treated within the prolonged time window, it should be underscored that it did not exceed the percentages reported by either the DAWN or DEFUSE-3 trial (6% and 7%, respectively) [5,6]. Another important fact is that the higher sICH rates were not translated into higher likelihood of disability or mortality at 3 months.

In a previous systematic review and meta-analysis conducted by our group, an analysis of pooled RCT and real-world data did not provide sufficient evidence regarding the efficacy of EVT in patients with BAO [9]. Importantly, this result was mostly driven by the included RCTs (RR: 1.21, 95% CI 0.96–1.53) in comparison to the observational data that favored the intervention (RR: 0.67, 95% CI 0.53–0.85) [9]. Observational cohort studies, mirroring clinical practice in a real-world setting, have reported a significant proportion of patients achieving good functional outcomes at 3 months, ranging from 37 to 46% [9,32,33]. Furthermore, our previous meta-analysis did not include the two recent RCTs published in 2022. In our previous work, by using conditional probability analysis, we showed that a RCT with a sample size of 100 patients could shift the overall effect of the meta-analysis to a statistically significant result. ATTENTION [12] and BAOCHE [13] had a combined patient population of 557, providing enough power for a significant result to be achieved. Yet, those two studies used more restrictive inclusion criteria and selected patients with a more favorable profile toward EVT-associated efficacy: prolonged treatment time window, younger patients, with minimum pre-stroke disability and without significant ischemic changes on baseline CT. Therefore, applicability of EVT in other cohorts than those selected by the included RCTs is still questionable.

Other systematic reviews and meta-analyses have also aimed to investigate the efficacy and safety of EVT plus BMT versus BMT alone [34,35,36,37,38,39]. One study provided a meta-analysis of pooled RCT and real-world data with inconclusive results [34], while another two studies included data from RCTs that were not published at that moment—but were presented during the European Stroke Organisation Conference (ESOC) 2022—resulting in significant uncertainty regarding quality assessment and critical interpretation of the results [35,36]. A pre-registered protocol was not available for two of the projects [35,36]. Stratification for IVT pre-treatment was not performed in the majority of the studies [34,35,36,38,39]. Finally, none of the existed projects provided an estimation for the robustness of the presented results following a fragility index meta-analysis.

Our present meta-analysis followed a prespecified protocol and included all available RCTs that have been completed and published to date, investigating EVT efficacy and safety of BAO patients. Additionally, it provided an estimate of robustness of the presented results while different potential sources of heterogeneity were addressed. Despite these strengths, several limitations of our study should also be acknowledged. First, other sources of heterogeneity could exist, considering the differences of the inclusion criteria among the studies, such as age, initial stroke severity, and time for stroke onset since randomization. Unfortunately, a meta-regression analysis with the aim of evaluating potential interference of these variables could not be performed, due to the limited number of studies included in the meta-analysis. An individual patient data meta-analysis, rather than a study-level meta-analysis, may explore these disparities and adjust for potential differences among the included patients, and therefore, a future collaboration could focus on such an effort. Apart from the observed heterogeneity, there were significant biases during quality assessment, especially due to deviations from intended interventions and absence of allocation concealment. Last and most important, the lack of robustness with regard to the association of EVT and practically all assessed efficacy and safety outcomes indicates caution in the interpretation of our findings.

## 5. Conclusions

In conclusion, the current meta-analysis showed that EVT combined with BMT was associated with a higher likelihood of achieving good functional outcome, functional independence and any functional improvement at 3 months compared to BMT alone. Despite the higher risk of any ICH and sICH among EVT-treated patients, 3-month mortality did not differ between the two groups. An individual patient data meta-analysis is warranted to further explore heterogeneity and to adjust for different potential confounders, providing further insight.

## Figures and Tables

**Table 1:** Characteristics of studies included in the systematic review and meta-analysis.

Study	Recruiting Centers	Period of Enrollment	Target Population	EVT and BMT		BMT Alone	
				N	Patients Characteristics	N	Patients Characteristics
ATTENTION [12]	China	February 2021–January 2022	BAO within 12 h, with NIHSS ≥ 10 and pre-stroke mRS and PC-ASPECTS selection stratified by age	226	Mean age: 66.0 ± 11.1 years; 66% males; median NIHSS: 24 (15–35); median PC-ASPECTS: 9 (8–10); median time from stroke onset to randomization: 5.1 (3.6–7.2) h; 31% with IVT pretreatment	114	Mean age: 67.3 ± 10.2 years; 72% males; median NIHSS: 24 (14–35); median PC-ASPECTS: 10 (8–10); median time from stroke onset to randomization: 4.9 (3.5–7.0) h; 34% with IVT pretreatment
BAOCHE [13]	China	August 2016–June 2021	BAO between 6 to 24 h, age ≤ 80, with NIHSS ≥ 10 (later expanded to ≥6), pre-stroke mRS ≤ 1, PC-ASPECTS ≥ 6	110	Mean age: 64.2 ± 9.6 years; 73% males; median NIHSS: 20 (15–29); median PC-ASPECTS: 8 (7–10); median time from stroke onset to randomization: 11.1 (8.5–14.4) h; 14% with IVT pretreatment	107	Mean age: 63.7 ± 9.8 years; 74% males; median NIHSS: 19 (12–30); median PC = ASPECTS: 8 (7–10); median time from stroke onset to randomization: 11.0 (8.2–14.0) h; 21% with IVT pretreatment
BASICS [10]	Interna tional	October 2011–December 2019	BAO within 6 h, age ≤ 85 (later expanded to >85), with NIHSS ≥ 10 (later expanded to <10), pre-stroke mRS ≤ 2	154	Mean age: 66.8 ± 13.1 years; 65% males; median NIHSS: 21 (NR); median PC-ASPECTS: 10 (10–10); median time from stroke onset to randomization: 4.4 (3.3–6.2) h *; 79% with IVT pretreatment	146	Mean age: 67.2 ± 11.9 years; 66% males; median NIHSS: 22 (NR); median PC-ASPECTS: 10 (10–10); median time from stroke onset to randomization: NR; 80% with IVT pretreatment
BEST [11]	China	April 2015–September 2017	BAO, within 8 h, pre-stroke mRS ≤ 2	66	Mean age: 62.0 ± 17.8 years; 73% males; median NIHSS: 32 (18–38); median PC-ASPECTS: 8 (7–9); median time from stroke onset to randomization: 4.1 (2.3–6) h; 27% with IVT pretreatment	65	Mean age: 65.7 ± 12.6 years; 80% males; median NIHSS: 26 (13–37); median PC-ASPECTS: 8 (7–9); median time from stroke onset to randomization: 4.6 (3.2–6.5) h; 32% with IVT pretreatment

EVT: endovascular treatment; BMT: best medical treatment; N: number; SD: standard deviation; mRS: modified Rankin scale; NIHSS: National Institutes Health Stroke Scale; IQR: interquartile range; PC-ASPECTS: posterior circulation Acute Stroke Prognosis Early CT Score; IVT: intravenous thrombolysis; BAO: basilar artery occlusion; NR: not reported. * In BASICS [10], time from stroke onset to randomization was not reported. In the table, time from stroke onset to endovascular treatment is presented to allow for an indirect comparison with other studies.

**Table 2:** Overview of analyses for primary and secondary outcomes.

Variable	Effect			Fragility Index	Interpretation
	N of Studies	Risk Ratio (95% CI)	I^2^, *p* for Cochran Q		
Primary Outcome					
Good Functional Outcome (mRS 0–3)	4	1.54 (1.16–2.05)	60%; 0.06	9	Fragile/Somewhat Robust
Secondary Efficacy Outcomes					
Functional Independence (mRS 0–2)	4	1.83 (1.08–3.08)	79%; 0.02	4	Highly Fragile/Not Robust
Reduced Disability	4	1.96 (1.26–3.05) *	59%; 0.06	NA	NA
Secondary Safety Outcomes					
Symptomatic Intracranial Hemorrhage	4	7.78 (2.36–25.61)	0%; 0.97	5	Fragile/Somewhat Robust
Any Intracranial Hemorrhage	4	2.85 (1.50–5.44)	16%; 0.31	5	Fragile/Somewhat Robust
All-cause mortality	4	0.76 (0.65–0.89)	0%; 0.42	9	Fragile/Somewhat Robust

mRS: modified Rankin Scale; CI: confidence interval; NA: not applicable. * Adjusted common OR.

## Data Availability

All data used for this study are provided in this article and its accompanying Supplementary Materials.

## Supplementary Materials

The following supporting information can be downloaded at: https://www.mdpi.com/article/10.3390/jcm12072617/s1, Figure S1: Forest plot presenting the pooled mean of age (in years) of the patients enrolled in either arm of the included randomized-controlled clinical trials.; Figure S2: Forest plot presenting the pooled proportion of men among the total participants in the included randomized-controlled clinical trials.; Figure S3: Forest plot presenting the pooled median of NIHSS among the patients enrolled in either arm of the included randomized-controlled clinical trials, as calculated by the quantile estimation (QE) method.; Figure S4: Forest plot presenting the pooled proportion of patients receiving pretreatment with intravenous thrombolysis (IVT) among the total participants in the included randomized-controlled clinical trials, after stratification for recruiting centers (limited to China versus international recruitment).; Figure S5: Summary plot presenting the quality assessment of included randomized controlled clinical trials using the Cochrane Collaboration tool (RoB 2).; Figure S6: Traffic Light Plot presenting the quality assessment of included randomized controlled clinical trials using the Cochrane Collaboration tool (RoB 2).; Figure S7: Forest plot presenting the association of endovascular treatment (EVT) combined with best medical treatment (BMT) compared to BMT alone with good functional outcomes (mRS 0-3) at 3 months among acute basilar artery occlusion patients, after stratification for recruiting centers (limited to China versus international recruitment).; Figure S8: Forest plot presenting the association of endovascular treatment (EVT) combined with best medical treatment (BMT) compared to BMT alone with good functional outcomes (mRS 0-3) at 3 months among acute basilar artery occlusion patients, after stratification for the administration of intravenous thrombolysis (IVT) and assessed by generic calculation of available Risk Ratios reported by the included studies.; Figure S9: Forest plot presenting the association of endovascular treatment (EVT) combined with best medical treatment (BMT) compared to BMT alone with good functional outcomes (mRS 0-3) at 3 months among acute basilar artery occlusion patients, after stratification for the administration of intravenous thrombolysis (IVT) and assessed by generic calculation of available Odds Ratios reported by the included studies.; Figure S10: Forest plot presenting the association of endovascular treatment (EVT) combined with best medical treatment (BMT) compared to BMT alone with functional independence (mRS 0-2) at 3 months among acute basilar artery occlusion patients.; Figure S11: Forest plot presenting the association of endovascular treatment (EVT) combined with best medical treatment (BMT) compared to BMT alone with functional independence (mRS 0-2) at 3 months among acute basilar artery occlusion patients, after stratification for recruiting centers (limited to China versus international recruitment). Figure S12: Forest plot presenting the pooled adjusted common odds ratio for reduced disability at 3 months (improvement of a least 1 point on the mRS) in patients with acute basilar artery occlusion treated with endovascular treatment (EVT) combined with best medical treatment (BMT) vs. BMT alone.; Figure S13: Forest plot presenting the pooled adjusted common odds ratio for reduced disability at 3 months (improvement of a least 1 point on the mRS) in patients with acute basilar artery occlusion treated with endovascular treatment (EVT) combined with best medical treatment (BMT) vs. BMT alone, after stratification for recruiting centers (limited to China versus international recruitment).; Figure S14: Forest plot presenting the pooled proportion of symptomatic intracranial hemorrhage (sICH) among patients with acute basilar artery occlusion treated with endovascular treatment (EVT) combined with best medical treatment (BMT).; Figure S15: Forest plot presenting the association of endovascular treatment (EVT) combined with best medical treatment (BMT) compared to BMT alone with symptomatic intracranial hemorrhage (sICH) among acute basilar artery occlusion patients, after stratification for recruiting centers (limited to China versus international recruitment).; Figure S16: Forest plot presenting the association of endovascular treatment (EVT) combined with best medical treatment (BMT) compared to BMT alone with any intracranial hemorrhage (aICH) among acute basilar artery occlusion patients.; Figure S17: Forest plot presenting the association of endovascular treatment (EVT) combined with best medical treatment (BMT) compared to BMT alone with any intracranial hemorrhage (aICH) among acute basilar artery occlusion patients, after stratification for recruiting centers (limited to China versus international recruitment).; Figure S18: Forest plot presenting the association of endovascular treatment (EVT) combined with best medical treatment (BMT) compared to BMT alone with mortality at 3 months among acute basilar artery occlusion patients, after stratification for recruiting centers (limited to China versus international recruitment). Table S1: Table of excluded studies with reasons for exclusion. References [40,41,42,43,44,45,46] are cited in Supplementary File.

## Trial Registration

The pre-specified protocol of the present systematic review and meta-analysis has been registered in the International Prospective Register of Ongoing Systematic Reviews PROSPERO (registration ID: CRD42022373387).
