# Supplementary material for: Endovascular Treatment for Acute Basilar Artery Occlusion: A Fragility Index Meta-Analysis
Source: J Clin Med. 2023 Mar 30;12(7):2617. doi: 10.3390/jcm12072617 (PMC10094975; doi:10.3390/jcm12072617)

Methods S1

Tables:

Table S1. Table of excluded studies with reasons for exclusion.

References used in the Supplement.

Figures:

Figures S1-S18

Methods S1

<u>Complete search algorithm used in MEDLINE search.</u>

(("basilar"[All Fields] OR "posterior"[All Fields]) AND ("mechanical thrombectomy"[All Fields] OR "endovascular"[All Fields]) AND "stroke"[All Fields]

<u>Complete search algorithm used in SCOPUS search.</u>

(ALL ({stroke}) AND ALL ({basilar} OR {posterior}) AND ALL ({mechanical thrombectomy} OR {endovascular}) AND ALL ({randomized controlled clinical trial} OR {randomized controlled trial} OR {RCT})) AND (LIMIT-TO (DOCTYPE , "ar"))

Table S1. Table of excluded studies with reasons for exclusion.

Study Name	Reason(s) for Exclusion
Li et al. [1]	Observational study
Pirson et al. [2]	Observational study
Schonewille et al. [3]	Editorial
Singer et al. [4]	Observational study
Tao et al. [5]	Observational study
Zi et al. [6]	Observational study

References used in the Supplement.

1. Li F, Sang H, Song J, Guo Z, Liu S, Luo W, et al. One-Year Outcome After Endovascular Treatment for Acute Basilar Artery Occlusion. Stroke. 2022;53(1):e9-e13.
2. Pirson FAV, Boodt N, Brouwer J, Bruggeman AAE, den Hartog SJ, Goldhoorn RB, et al. Endovascular Treatment for Posterior Circulation Stroke in Routine Clinical Practice: Results of the Multicenter Randomized Clinical Trial of Endovascular Treatment for Acute Ischemic Stroke in the Netherlands Registry. Stroke. 2022;53(3):758-68.
3. Schonewille WJ. Favorable Outcomes in Endovascular Therapy for Basilar-Artery Occlusion. The New England journal of medicine. 2022;387(15):1428-9.
4. Singer OC, Berkefeld J, Nolte CH, Bohner G, Haring HP, Trenkler J, et al. Mechanical recanalization in basilar artery occlusion: the ENDOSTROKE study. Annals of neurology. 2015;77(3):415-24.
5. Tao C, Qureshi AI, Yin Y, Li J, Li R, Xu P, et al. Endovascular Treatment Versus Best Medical Management in Acute Basilar Artery Occlusion Strokes: Results From the ATTENTION Multicenter Registry. Circulation. 2022;146(1):6-17.
6. Zi W, Qiu Z, Wu D, Li F, Liu H, Liu W, et al. Assessment of Endovascular Treatment for Acute Basilar Artery Occlusion via a Nationwide Prospective Registry. JAMA neurology. 2020;77(5):561-73.
7. McGrath S, Sohn H, Steele R, Benedetti A. Meta-analysis of the difference of medians. 2020;62(1):69-98.
8. Sterne JAC, Savović J, Page MJ, Elbers RG, Blencowe NS, Boutron I, et al. RoB 2: a revised tool for assessing risk of bias in randomised trials. BMJ (Clinical research ed). 2019;366:l4898.

Figure S1. Forest plot presenting the pooled mean of age (in years) of the patients enrolled in either arm of the included randomized-controlled clinical trials.

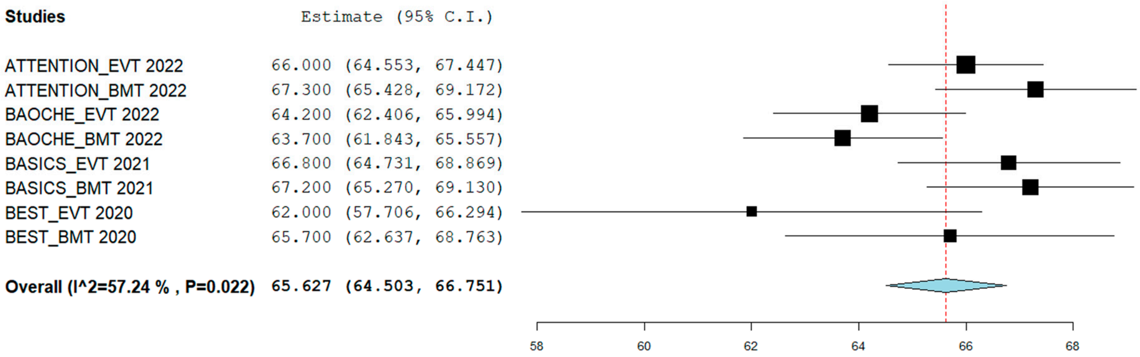

Figure S2. Forest plot presenting the pooled proportion of men among the total participants in the included randomized-controlled clinical trials.

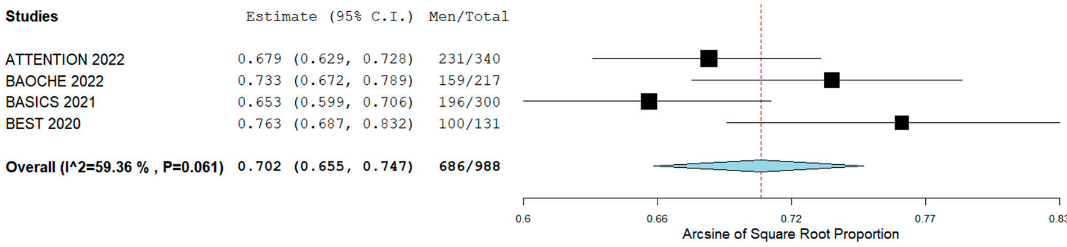

Figure S3. Forest plot presenting the pooled median of NIHSS among the patients enrolled in either arm of the included randomized-controlled clinical trials, as calculated by the quantile estimation (QE) method.[7]

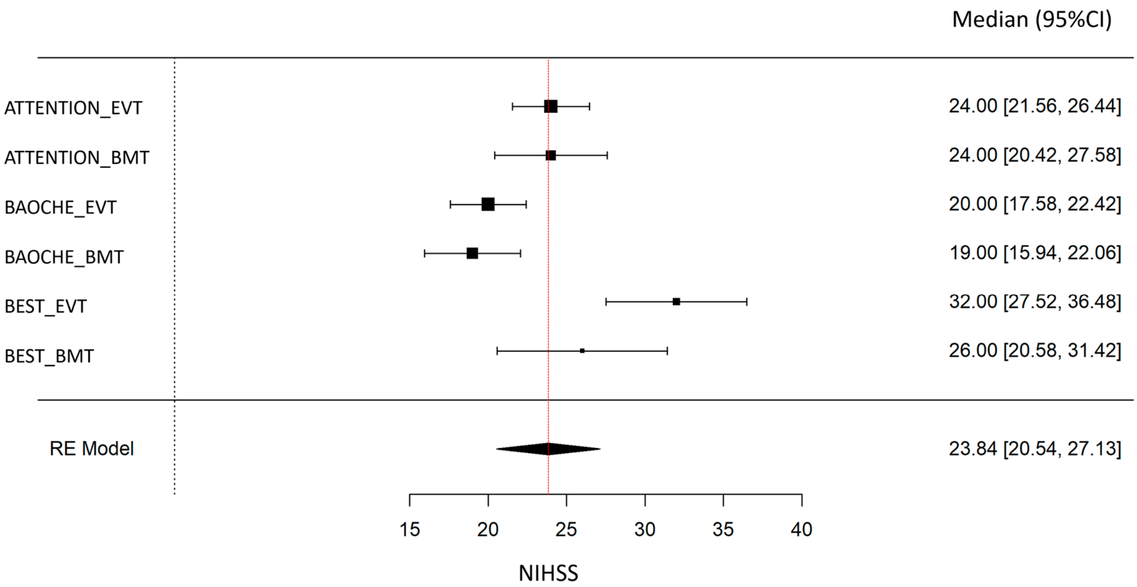

Figure S4. Forest plot presenting the pooled proportion of patients receiving pretreatment with intravenous thrombolysis (IVT) among the total participants in the included randomized-controlled clinical trials, after stratification for recruiting centers (limited to China versus international recruitment).

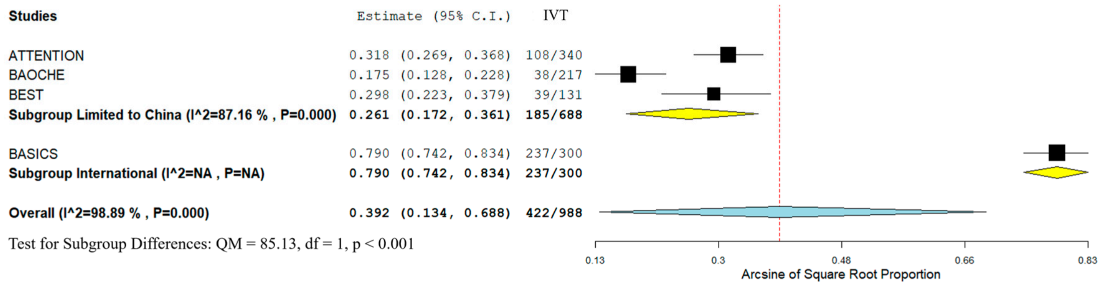

Figure S5. Summary plot presenting the quality assessment of included randomized controlled clinical trials using the Cochrane Collaboration tool (RoB 2).[8]

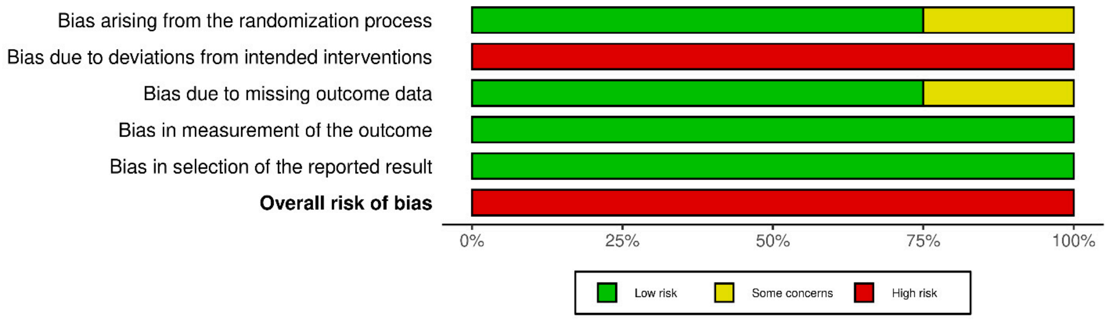

Figure S6. Traffic Light Plot presenting the quality assessment of included randomized controlled clinical trials using the Cochrane Collaboration tool (RoB 2).[8]

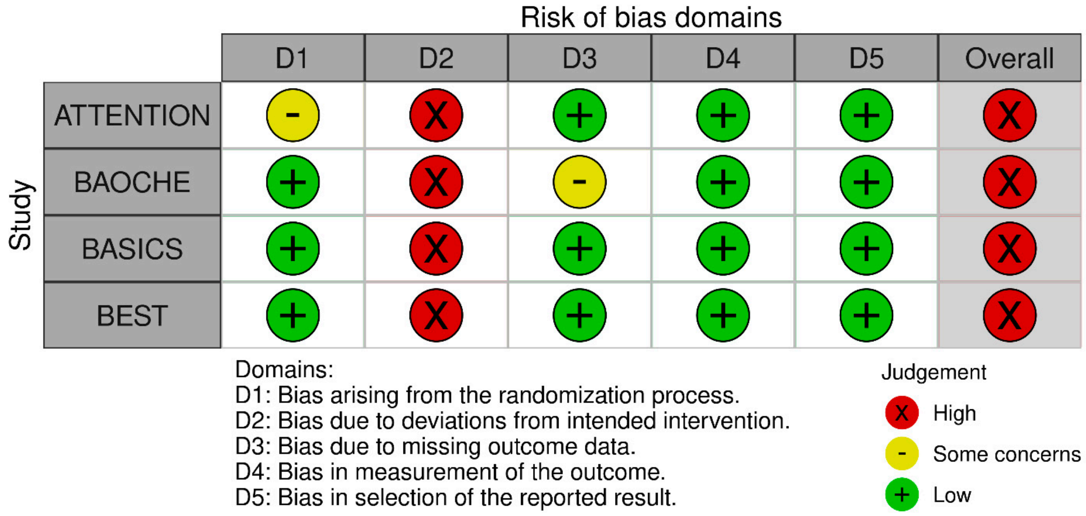

Figure S7: Forest plot presenting the association of endovascular treatment (EVT) combined with best medical treatment (BMT) compared to BMT alone with good functional outcomes (mRS 0-3) at 3 months among acute basilar artery occlusion patients, after stratification for recruiting centers (limited to China versus international recruitment).

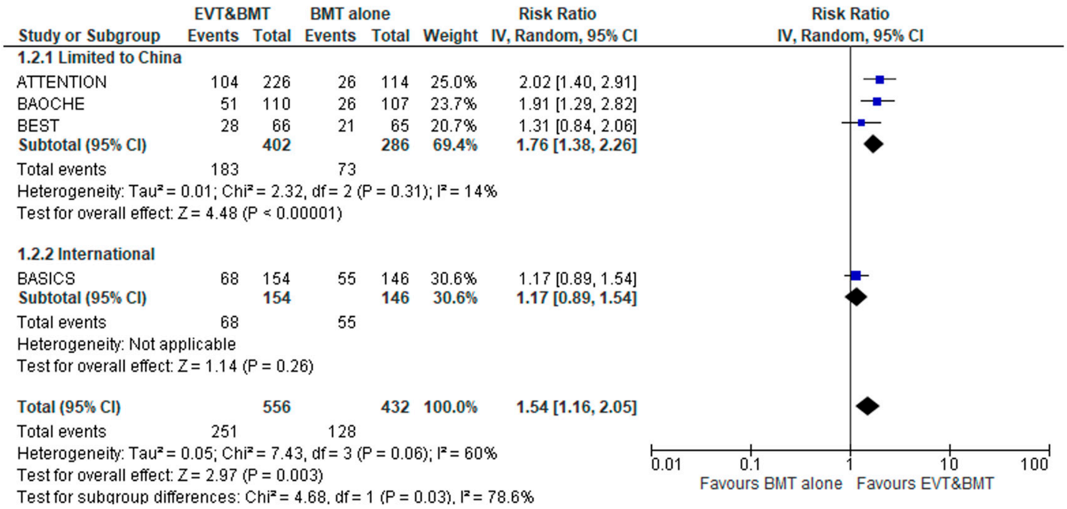

Figure S8: Forest plot presenting the association of endovascular treatment (EVT) combined with best medical treatment (BMT) compared to BMT alone with good functional outcomes (mRS 0-3) at 3 months among acute basilar artery occlusion patients, after stratification for the administration of intravenous thrombolysis (IVT) and assessed by generic calculation of available Risk Ratios reported by the included studies.

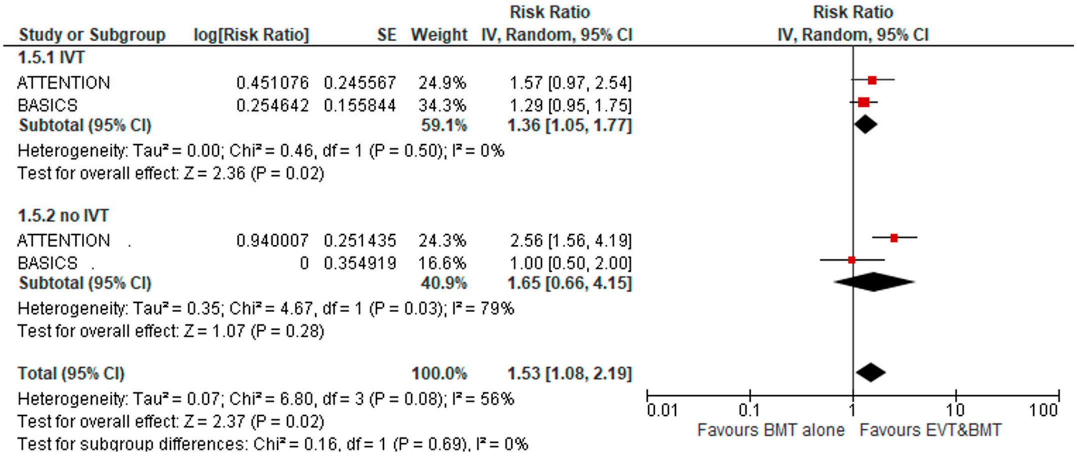

Figure S9: Forest plot presenting the association of endovascular treatment (EVT) combined with best medical treatment (BMT) compared to BMT alone with good functional outcomes (mRS 0-3) at 3 months among acute basilar artery occlusion patients, after stratification for the administration of intravenous thrombolysis (IVT) and assessed by generic calculation of available Odds Ratios reported by the included studies.

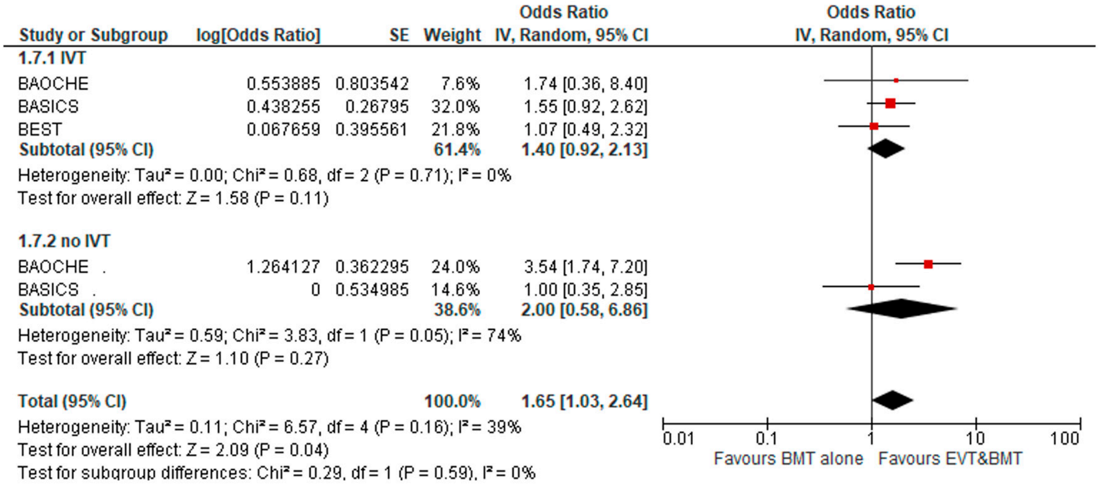

Figure S10: Forest plot presenting the association of endovascular treatment (EVT) combined with best medical treatment (BMT) compared to BMT alone with functional independence (mRS 0-2) at 3 months among acute basilar artery occlusion patients.

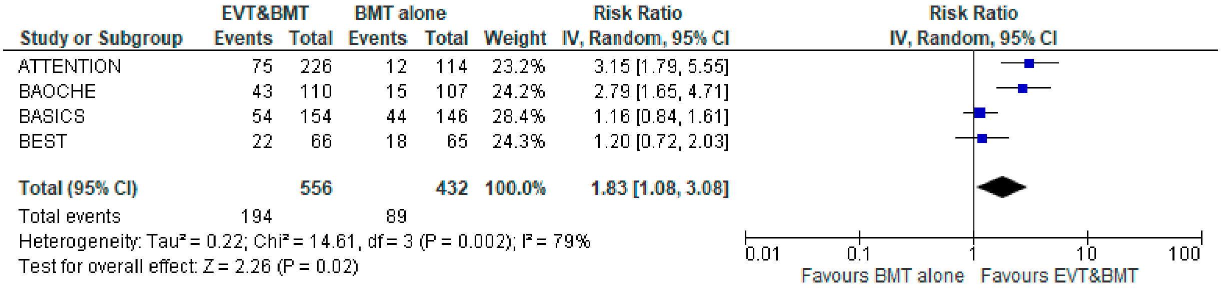

Figure S11: Forest plot presenting the association of endovascular treatment (EVT) combined with best medical treatment (BMT) compared to BMT alone with functional independence (mRS 0-2) at 3 months among acute basilar artery occlusion patients, after stratification for recruiting centers (limited to China versus international recruitment).

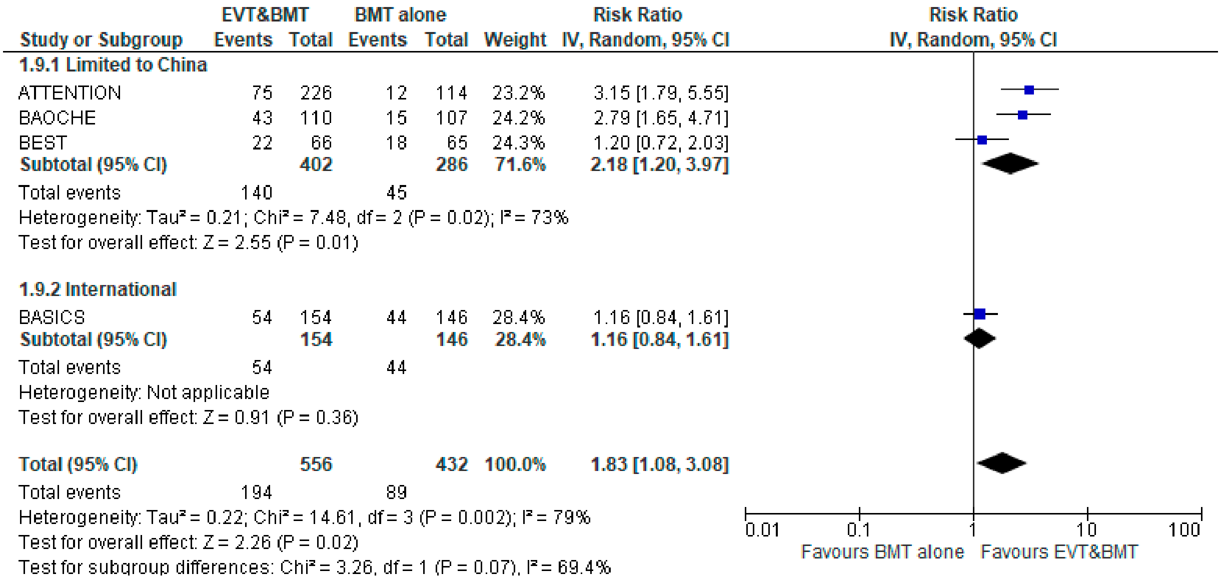

Figure S12: Forest plot presenting the pooled adjusted common odds ratio for reduced disability at 3 months (improvement of a least 1 point on the mRS) in patients with acute basilar artery occlusion treated with endovascular treatment (EVT) combined with best medical treatment (BMT) vs. BMT alone.

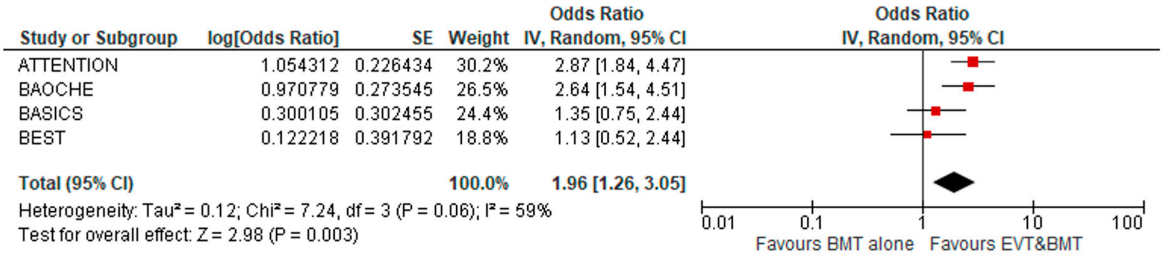

Figure S13: Forest plot presenting the pooled adjusted common odds ratio for reduced disability at 3 months (improvement of a least 1 point on the mRS) in patients with acute basilar artery occlusion treated with endovascular treatment (EVT) combined with best medical treatment (BMT) vs. BMT alone, after stratification for recruiting centers (limited to China versus international recruitment).

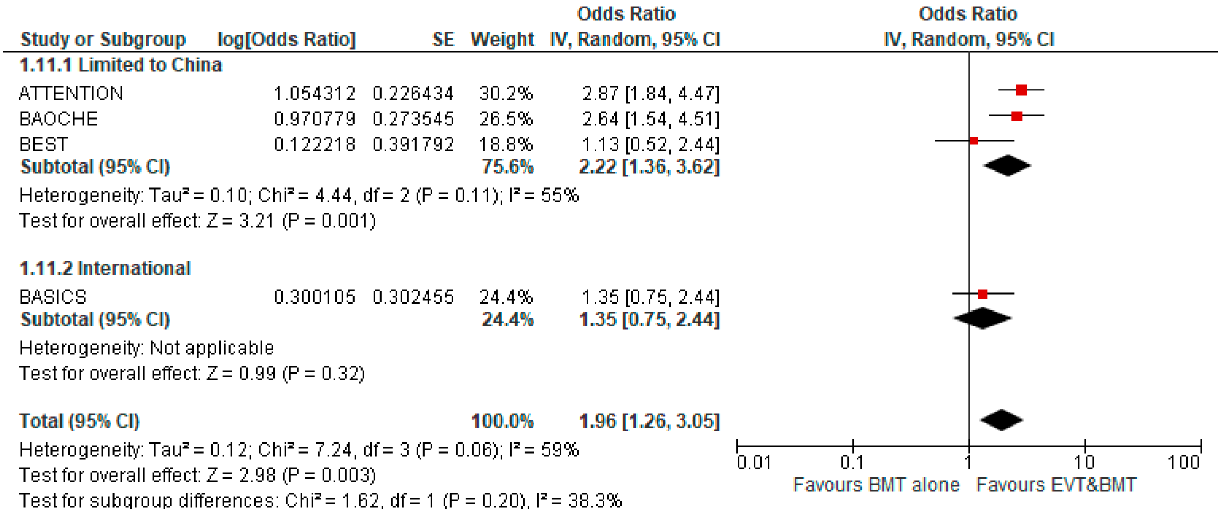

Figure S14: Forest plot presenting the pooled proportion of symptomatic intracranial hemorrhage (sICH) among patients with acute basilar artery occlusion treated with endovascular treatment (EVT) combined with best medical treatment (BMT).

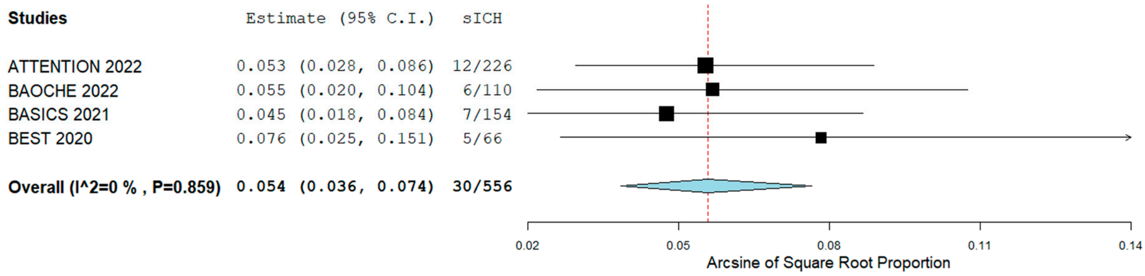

Figure S15: Forest plot presenting the association of endovascular treatment (EVT) combined with best medical treatment (BMT) compared to BMT alone with symptomatic intracranial hemorrhage (sICH) among acute basilar artery occlusion patients, after stratification for recruiting centers (limited to China versus international recruitment).

	EVT&BMT		BMT alone			Risk Ratio
**Study or Subgroup**	**Events**	**Total**	**Events**	**Total**	**Weight**	**IV, Random, 95% CI**
**1.13.1 Limited to China**						
ATTENTION	12	226	0	114	17.9%	12.67 [0.76, 212.01]
BAOCHE	6	110	1	107	32.2%	5.84 [0.71, 47.67]
BEST	5	66	0	65	17.2%	10.84 [0.61, 192.08]
**Subtotal (95% CI)**		**402**		**286**	**67.3%**	**8.40 [1.96, 35.92]**
Total events	23		1			
Heterogeneity: Tau² = 0.00; Chi² = 0.23, df = 2 (P = 0.89); I² = 0%						
Test for overall effect: Z = 2.87 (P = 0.004)						
**1.13.2 International**						
BASICS	7	154	1	146	32.7%	6.64 [0.83, 53.28]
**Subtotal (95% CI)**		**154**		**146**	**32.7%**	**6.64 [0.83, 53.28]**
Total events	7		1			
Heterogeneity: Not applicable						
Test for overall effect: Z = 1.78 (P = 0.07)						
**Total (95% CI)**		**556**		**432**	**100.0%**	**7.78 [2.36, 25.61]**
Total events	30		2			
Heterogeneity: Tau² = 0.00; Chi² = 0.26, df = 3 (P = 0.97); I² = 0%						
Test for overall effect: Z = 3.37 (P = 0.0007)						
Test for subgroup differences: Chi² = 0.03, df = 1 (P = 0.86), I² = 0%						

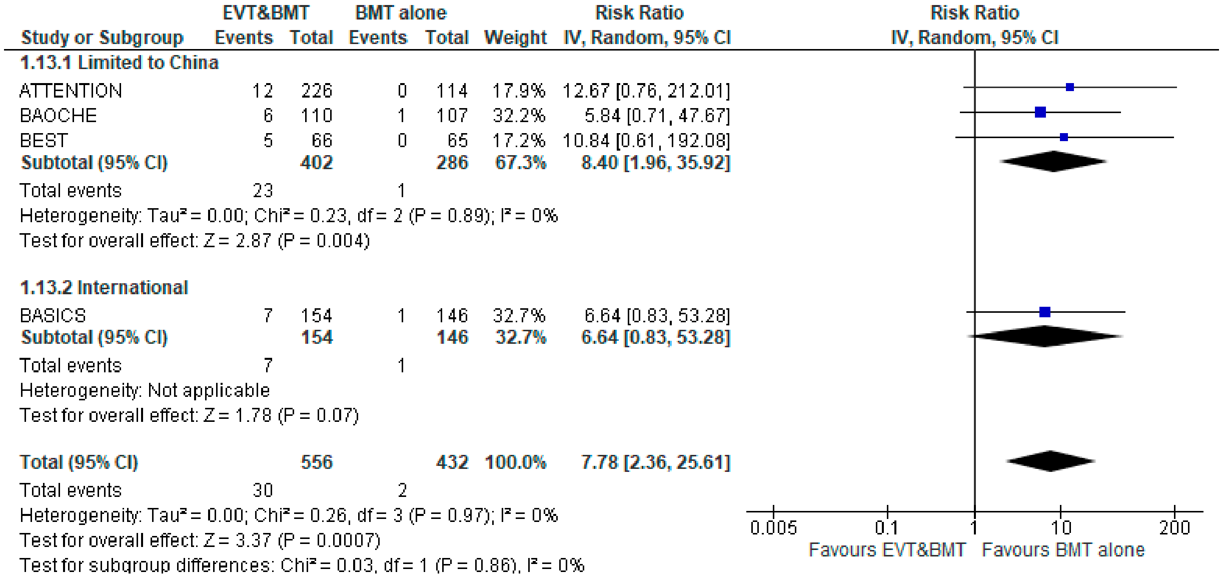

Figure S16: Forest plot presenting the association of endovascular treatment (EVT) combined with best medical treatment (BMT) compared to BMT alone with any intracranial hemorrhage (aICH) among acute basilar artery occlusion patients.

	EVT&BMT		BMT alone			Risk Ratio
**Study or Subgroup**	**Events**	**Total**	**Events**	**Total**	**Weight**	**IV, Random, 95% CI**
ATTENTION	31	226	2	114	18.3%	7.82 [1.90, 32.09]
BAOCHE	14	110	4	107	28.9%	3.40 [1.16, 10.01]
BASICS	12	154	7	146	38.1%	1.63 [0.66, 4.01]
BEST	5	66	2	65	14.6%	2.46 [0.50, 12.24]
**Total (95% CI)**		**556**		**432**	**100.0%**	**2.85 [1.50, 5.44]**
Total events	62		15			
Heterogeneity: Tau² = 0.07; Chi² = 3.57, df = 3 (P = 0.31); I² = 16%						
Test for overall effect: Z = 3.19 (P = 0.001)						

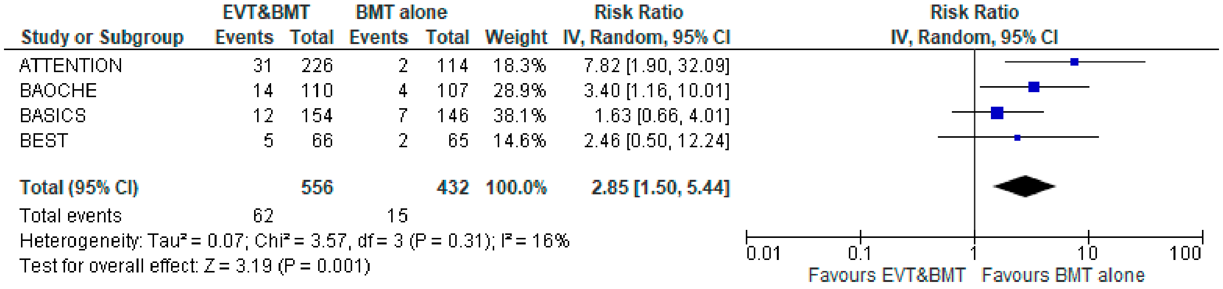

Figure S17: Forest plot presenting the association of endovascular treatment (EVT) combined with best medical treatment (BMT) compared to BMT alone with any intracranial hemorrhage (aICH) among acute basilar artery occlusion patients, after stratification for recruiting centers (limited to China versus international recruitment).

	EVT&BMT		BMT alone			Risk Ratio
**Study or Subgroup**	**Events**	**Total**	**Events**	**Total**	**Weight**	**IV, Random, 95% CI**
**1.15.1 Limited to China**						
ATTENTION	31	226	2	114	18.3%	7.82 [1.90, 32.09]
BAOCHE	14	110	4	107	28.9%	3.40 [1.16, 10.01]
BEST	5	66	2	65	14.6%	2.46 [0.50, 12.24]
**Subtotal (95% CI)**		**402**		**286**	**61.9%**	**4.02 [1.89, 8.56]**
Total events	50		8			
Heterogeneity: Tau² = 0.00; Chi² = 1.30, df = 2 (P = 0.52); I² = 0%						
Test for overall effect: Z = 3.61 (P = 0.0003)						
**1.15.2 International**						
BASICS	12	154	7	146	38.1%	1.63 [0.66, 4.01]
**Subtotal (95% CI)**		**154**		**146**	**38.1%**	**1.63 [0.66, 4.01]**
Total events	12		7			
Heterogeneity: Not applicable						
Test for overall effect: Z = 1.05 (P = 0.29)						
**Total (95% CI)**		**556**		**432**	**100.0%**	**2.85 [1.50, 5.44]**
Total events	62		15			
Heterogeneity: Tau² = 0.07; Chi² = 3.57, df = 3 (P = 0.31); I² = 16%						
Test for overall effect: Z = 3.19 (P = 0.001)						
Test for subgroup differences: Chi² = 2.27, df = 1 (P = 0.13), I² = 55.9%						

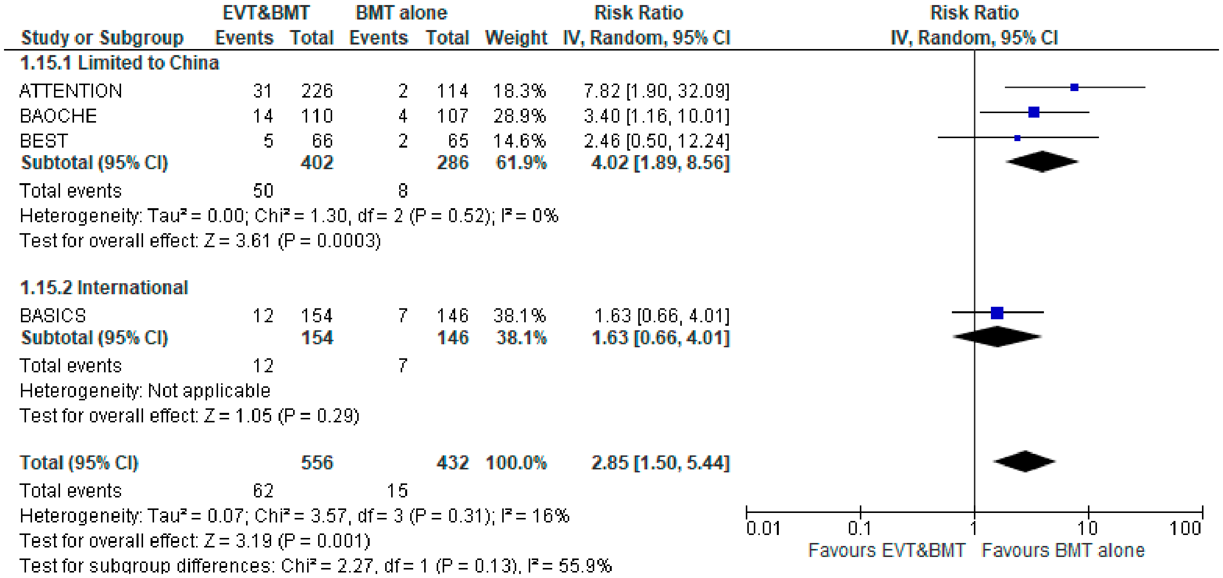

Figure S18: Forest plot presenting the association of endovascular treatment (EVT) combined with best medical treatment (BMT) compared to BMT alone with mortality at 3 months among acute basilar artery occlusion patients, after stratification for recruiting centers (limited to China versus international recruitment).

	EVT&BMT		BMT alone			Risk Ratio
**Study or Subgroup**	**Events**	**Total**	**Events**	**Total**	**Weight**	**IV, Random, 95% CI**
**1.17.1 Limited to China**						
ATTENTION	83	226	63	114	40.5%	0.66 [0.52, 0.84]
BAOCHE	34	110	45	107	18.0%	0.73 [0.51, 1.05]
BEST	22	66	25	65	10.9%	0.87 [0.55, 1.37]
**Subtotal (95% CI)**		**402**		**286**	**69.4%**	**0.71 [0.59, 0.85]**
Total events	139		133			
Heterogeneity: Tau² = 0.00; Chi² = 1.06, df = 2 (P = 0.59); I² = 0%						
Test for overall effect: Z = 3.68 (P = 0.0002)						
**1.17.2 International**						
BASICS	59	154	63	146	30.6%	0.89 [0.68, 1.17]
**Subtotal (95% CI)**		**154**		**146**	**30.6%**	**0.89 [0.68, 1.17]**
Total events	59		63			
Heterogeneity: Not applicable						
Test for overall effect: Z = 0.85 (P = 0.39)						
**Total (95% CI)**		**556**		**432**	**100.0%**	**0.76 [0.65, 0.89]**
Total events	198		196			
Heterogeneity: Tau² = 0.00; Chi² = 2.81, df = 3 (P = 0.42); I² = 0%						
Test for overall effect: Z = 3.53 (P = 0.0004)						
Test for subgroup differences: Chi² = 1.76, df = 1 (P = 0.19), I² = 43.0%						

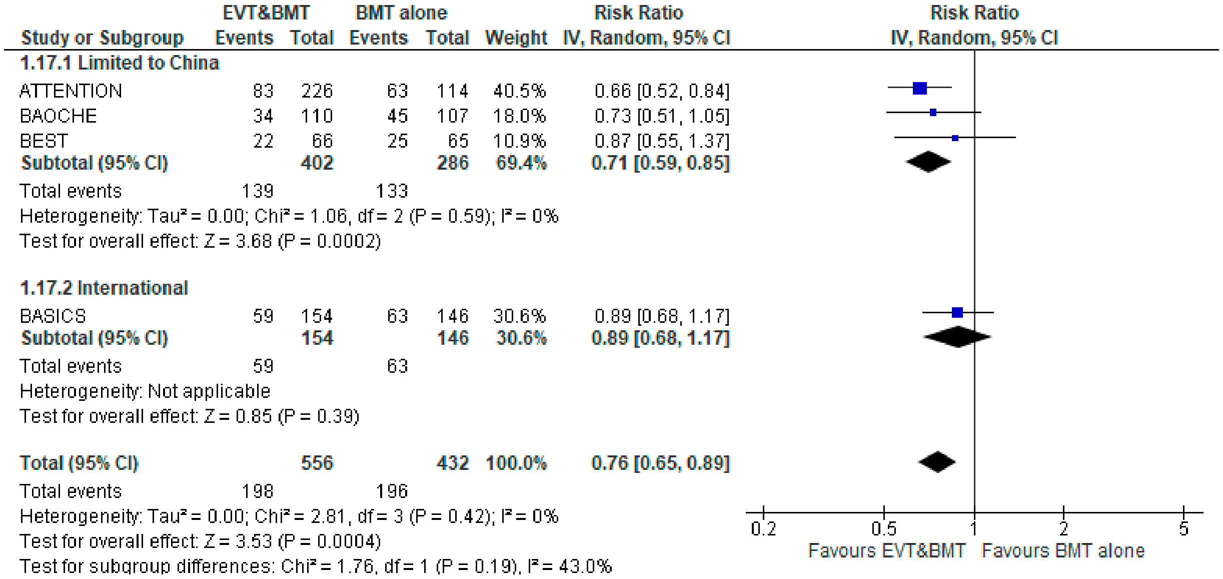

Supplement: Supplementary file 1 [file jcm-12-02617-s001.zip › jcm-2291021-supplementary.pdf]
